# Microarray-based method for screening of immunogenic proteins from bacteria

**DOI:** 10.1186/1477-3155-10-12

**Published:** 2012-03-21

**Authors:** Sebastian Hoppe, Frank F Bier, Markus von Nickisch-Rosenegk

**Affiliations:** 1 Fraunhofer Institute for Biomedical Engineering, Branch Potsdam, Am Mühlenberg 13, 14476 Potsdam, Germany; 2 University of Potsdam, Institute of Biochemistry and Biology, Potsdam, Germany

## Abstract

**Background:**

Detection of immunogenic proteins remains an important task for life sciences as it nourishes the understanding of pathogenicity, illuminates new potential vaccine candidates and broadens the spectrum of biomarkers applicable in diagnostic tools. Traditionally, immunoscreenings of expression libraries via polyclonal sera on nitrocellulose membranes or screenings of whole proteome lysates in 2-D gel electrophoresis are performed. However, these methods feature some rather inconvenient disadvantages. Screening of expression libraries to expose novel antigens from bacteria often lead to an abundance of false positive signals owing to the high cross reactivity of polyclonal antibodies towards the proteins of the expression host. A method is presented that overcomes many disadvantages of the old procedures.

**Results:**

Four proteins that have previously been described as immunogenic have successfully been assessed immunogenic abilities with our method. One protein with no known immunogenic behaviour before suggested potential immunogenicity.

We incorporated a fusion tag prior to our genes of interest and attached the expressed fusion proteins covalently on microarrays. This enhances the specific binding of the proteins compared to nitrocellulose. Thus, it helps to reduce the number of false positives significantly. It enables us to screen for immunogenic proteins in a shorter time, with more samples and statistical reliability. We validated our method by employing several known genes from *Campylobacter jejuni *NCTC 11168.

**Conclusions:**

The method presented offers a new approach for screening of bacterial expression libraries to illuminate novel proteins with immunogenic features. It could provide a powerful and attractive alternative to existing methods and help to detect and identify vaccine candidates, biomarkers and potential virulence-associated factors with immunogenic behaviour furthering the knowledge of virulence and pathogenicity of studied bacteria.

## Background

*Campylobacter jejuni *is one of the principal causing agents of bacterial gastroenteritis in industrialized countries [1]. From January to mid September 2011 52.940 *Campylobacter *infections have occurred in Germany alone [2]. Although infections predominantly result in mild self-limiting gastroenteritis, in some cases severe post-infection ramifications have been reported with the Guillain-Barré syndrome the major contributor [3]. The main route of infection and transmission is believed to be the incorrect handling and incomplete cooking of poultry. In fact, studies mention 62% of poultry carcasses to be contaminated with *Campylobacter *after slaughter [4].

On account of its widespread occurrence and clinical relevance testing for contamination of meat or the presence of *Campylobacter *infections in patients is necessary. Although several genomic typing methods exist [5,6] these are often time-consuming and laborious. In comparison, a rapid point-of-care device would need a more direct approach like the presence of antigens which react swiftly with specific antibodies. For many pathogens several different methods based on the antigen-antibody-reaction are already commercially available, e.g. Latex-Agglutination-Tests [7]. However, the knowledge of antigens for pathogens is often limited. Traditionally, testing for immunogenic proteins has been carried out by screening of expression libraries using nitrocellulose membranes [8]. Despite its simplicity, this method has a key shortcoming regarding bacterial proteins. The elucidation of novel antigens is extremely difficult as a result of the high cross-reactivity of polyclonal antibodies against the host bacterium of the library [9]. Therefore, a high number of false positives occur, which hinder a fast and successful search for new antigens.

With the emergence of fusion tags, protein purification has become more convenient. Several tags have been established and frequently used in all kinds of applications. Apart from other commonly used tags such as GST [10,11], MBP [12,13] or 6xHis [14,15] the HaloTag^® ^(Promega) features several unique characteristics. The HaloTag^® ^and its ligand bind covalently, leading to a strong, irreversible bond [16]. Further, it increases the amount of soluble protein expressed in contrast to other tags [17], as it reduces the formation of inclusion bodies during recombinant protein expression.

**Figure 1:**
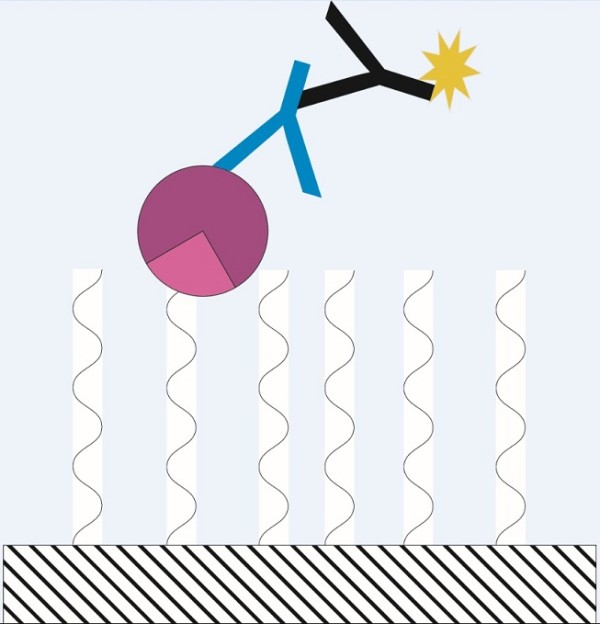
**Detection of immunogenic proteins on microarrays**. The cell lysates are spotted onto HaloLink™ slides (shaded area) with immobilized ligand (curved lines). The HaloTag^® ^(pink sector) is fused to the N-terminus of the protein of interest (purple sector) and attaches covalently to the ligand. Thus, the protein of interest is covalently attached to the surface prior to screening, whereas other proteins from the cell lysate are unbound and washed away after the coupling reaction. Subsequently, primary antibodies (blue) bind to the protein of interest. Afterwards, secondary antibodies (black) conjugated with a fluorophore (yellow) bind to the respective primary antibody. The fluorescent dye is used for detection of positive spots identifying sites of potential immunogenic proteins in the process.

**Figure 2:**
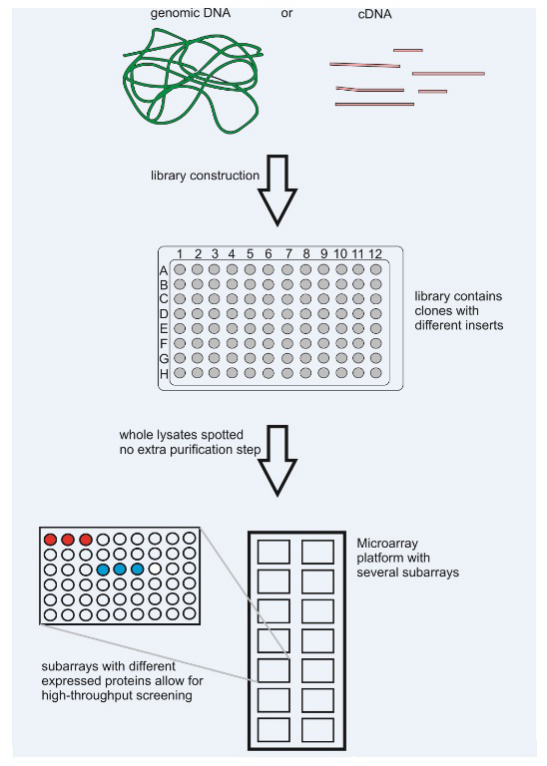
**Advantages of microarray platform for screening**. The method could readily be adapted to screen large expression libraries either from a cDNA or genomic source. Clones carrying different inserts are assigned unique positions in microtiter plates. Subsequently clones are cultivated, protein expression is induced and finally cells are lysed. The whole lysates can directly be spotted to the HaloLink™ Arrays rendering additional time-consuming and costly purification steps obsolete. As only fusion proteins will bind to the ligand, remaining proteins from the lysates are simply washed off after spotting minimizing background and cross-reactivity during the subsequent screening steps. The microarray platform allows for several thousand spots per slide allowing for easy high-throughput screening applications.

In this paper, we describe a method to covalently attach different HaloTag^® ^fusion proteins on HaloLink™ slides (see Figure 1) and consequently perform an immunoscreening using polyclonal antibodies in a microarray format, which is a suitable method for high-throughput applications such as screening of entire expression libraries (see Figure 2). This method reduces the pitfall of cross-reactive signals normally encountered with screenings and leads to a more rapid detection of immunogenic proteins compared to conventional methods.

Furthermore we show the outstanding performance of this method by expressing and detecting several proteins from *Campylobacter jejuni *previously described as immunogenic. As controls of the screening process, we included proteins from *Campylobacter *which have not been described as immunogenic before.

## Results

**Figure 3:**
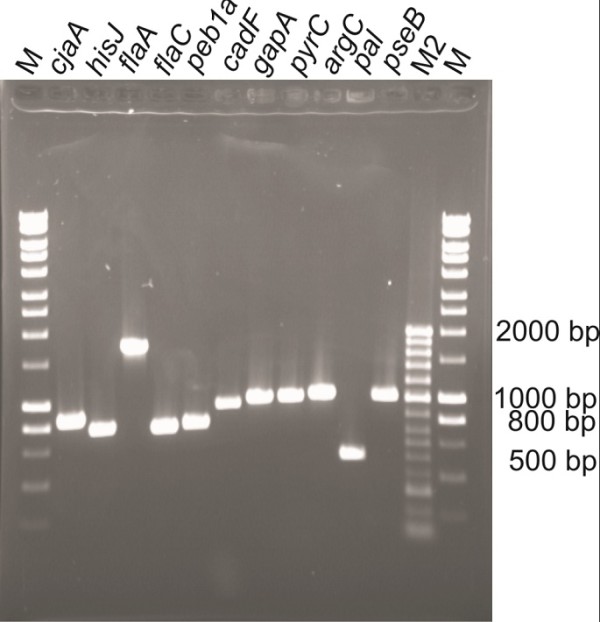
**Agarose gel of PCR products after amplification of *Campylobacter jejuni* genes from genomic DNA**. The band sizes match the respective length of each gene. Refer to Table 2 for expected gene lengths. As markers Hyper Ladder I (M) and II (M2) were used both supplied by Bioline.

We successfully amplified all but one gene (*cfrA*) from the genomic DNA of *Camyplobacter jejuni*. All eleven genes that were successfully amplified showed the correct length (see Figure 3) and were subsequently cloned to KRX single-step competent cells. The correct insert size was determined by Colony PCR. Plasmids from clones containing the correct-sized inserts were isolated and the MCS was sequenced using both a forward and a reverse primer (HT7 For and Flexi R). During cloning an extra GTT, which encodes for a valine residue in the proteins' primary structures, was inserted immediately prior to each stop codon. As a universal stop codon TAA was used, replacing other stop codons if present. This was mainly done to gain maximum flexibility during cloning as the Flexi vector system enables the direct transfer to other vectors with different tags. A GTT is mandatory to later transfer the gene of interest to a vector encoding a C-terminal tag.

For that reason, no 100% identity can be observed, when comparing the sequencing results with the original gene sequences from EMBL using a global alignment with free-end gaps. Nine of the plasmids differed only in the additional GTT mentioned above. For *pyrC *the usual stop codon TGA was replaced by TAA causing minor discrepancy in the alignment. The longest gene under investigation, *flaA *demonstrated the least conformity to the respective sequence from EMBL. The length of each sequence reaction was approximately 1300 nucleotides. Due to the numerous alterations in the sequence causing a shift in amino acid sequence, flaA was omitted from future investigation.

The correct expression of the encoded fusion proteins was assessed by SDS-Page. Analyzing the gel under fluorescent conditions reveals protein bands which have the HaloTag^® ^ligand attached. The PageRuler Plus prestained Protein ladder possesses two fluorescent bands, at 25 and 70 kDa respectively. HaloTag^® ^Standard Protein with a size of 60 kDa was also analyzed and helps as an additional size reference. The HaloTag^® ^features a size of 34 kDa alone.

For ease of use and brevity the expressed proteins are abbreviated and referred to as their corresponding gene names in plain text, e.g. pseB as the protein encoded by the gene *pseB*. Full protein names for each gene as taken from the KEGG database can be found in Table 1.

**Table 1:** Proteins encoded by genes used in this study.

Gene	Protein	Abbreviation
*cjaA*	putative amino-acid transporter periplasmic solute-binding protein	cjaA

*hisJ*	histidine-binding protein precursor	hisJ

*pal*	peptidoglycan associated lipoprotein	pal

*cfrA*	ferric enterobactin uptake receptor	cfrA

*flaC*	flagellin	flaC

*flaA*	flagellin	flaA

*peb1a*	bifunctional adhesin/ABC transporter aspartate/glutamate-binding protein	peb1a

*cadF*	OmpA-OmpF porin	cadF

*pyrC*	dihydroorotase (EC:3.5.2.3)	pyrC

*pseB*	UDP-GlcNAc-specific C4,6 dehydratase/C5 epimerase	pseB

*gapA*	glyceraldehyde 3-phosphate dehydrogenase (EC:1.2.1.12)	gapA

*argC*	N-acetyl-gamma-glutamyl-phosphate reductase (EC:1.2.1.38)	argC

The full names of each protein investigated are given according to the KEGG database with their coding genes. The gene names were used as abbreviations for the proteins in this paper

**Figure 4:**
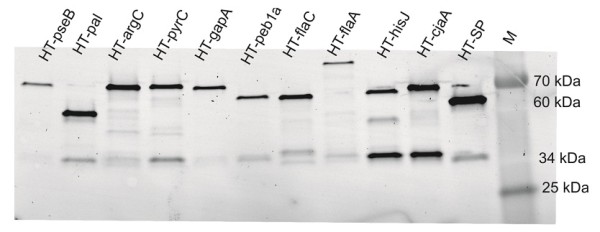
**SDS-PAGE of HaloTag^® ^fusion proteins incubated with HaloTag^® ^Alexa Fluor 488 ligand**. M refers to PageRuler Plus prestained protein ladder (Fermentas) with fluorescent bands at 70 kDa and 25 kDa, The HaloTag^® ^standard protein (HT-SP) was added as an additional size reference at 60 kDa. The bands match the expected sizes for each fusion protein. Additionally small fragments of only HaloTag^® ^(34 kDa) are visible which might be due to early-terminated translation.

Most of the fusion proteins investigated fall into a range between 61 and 73 kDa, namely HaloTag^® ^fused to argC (73 kDa), pyrC (72 kDa), pseB (71 kDa), gapA (70 kDa), cjaA (65 kDa), peb1 (62 kDa), hisJ (62 kDa) and flaC (61 kDa). Outside of this size range, only HaloTag^®^-flaA (93 kDa) and the small HaloTag^®^-pal (52 kDa) are found. For each protein, bands with the correct size could be detected, see Figure 4. Additionally, bands of smaller size are visible (34 kDA) which might be due to untimely termination of translation, potentially comprising only the HaloTag^®^, which features the corresponding size.

**Figure 5:**
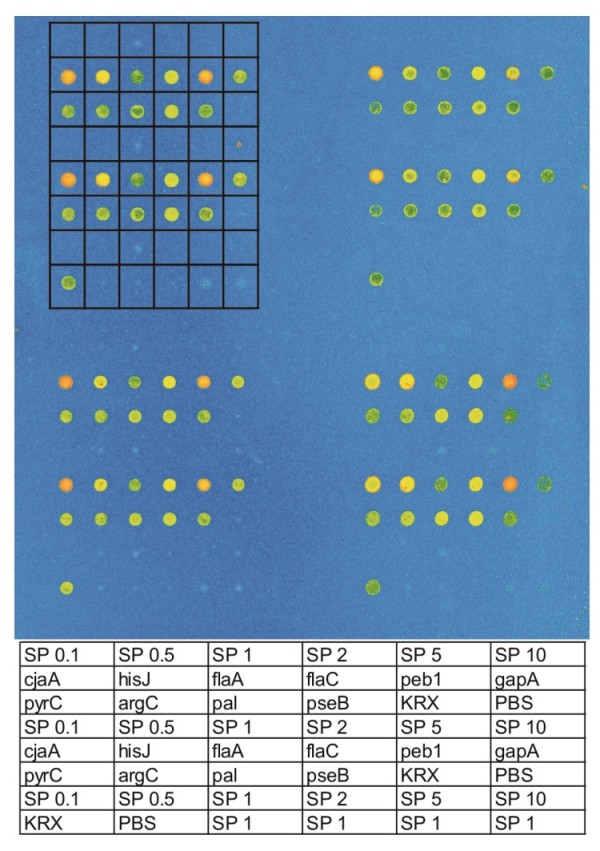
**Spotting layout**. The rectangular matrix within the picture marks one subarray. The corresponding samples are presented at the bottom. SP is the abbreviation for HaloTag^® ^standard protein with the number referring to the concentration of standard protein in the spotted solution in μg/ml. The gene names refer to the locations were corresponding fusion protein was released, KRX is cell extract without any fusion protein and PBS represents the buffer control. Each sample was spotted at least in duplicate.

The cell lysates containing the expressed fusion proteins were spotted to HaloLink™ Slides (Promega) by the QArray2 microarray spotter. For the spotting layout see Figure 5.

The immunogenicity of the immobilized proteins was assessed using polyclonal antibodies raised against whole and partially lysed attenuated cells of *Campylobacter jejuni*. Secondary antibodies conjugated with a fluorophore were used to detect signals. For better comparability the raw data was processed and contrast values were calculated.

**Figure 6:**
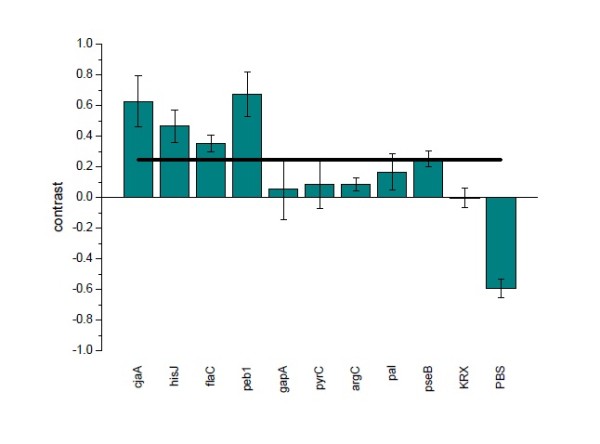
**Contrast value for one microarray slide**. The bars represent the median. The standard deviation (SD) is accounted for by the error bars. KRX represents cell lysate without HaloTag^® ^protein and PBS was spotted as a buffer control. The cutoff is represented by a bold horizontal line. Four proteins namely cjaA, hisJ, flaC and peb1a are above the cutoff. In comparison, gapA, pyrC and argC lie significantly below the cutoff value. Two proteins - pal and pseB - are approximately at the cutoff. The contrasts for KRX and PBS are at or below zero.

Figure 6 shows the resulting bar chart of the processed data of one slide. Four proteins showed contrast values above the cutoff. The contrast values ± s.d. for each of these proteins were 0.63 ± 0.17 (cjaA), 0.47 ± 0.10 (hisJ), 0.35 ± 0.05 (flaC) and 0.68 ± 0.15 (peb1a) respectively. In comparison, the contrast values of the following proteins were significantly below the cutoff: 0.06 ± 0.20 (gapA), 0.08 ± 0.15 (pyrC) and 0.09 ± 0.04 (argC). The last two proteins - pal and pseB - led to contrast values of 0.17 ± 0.11 (pal) and 0.25 ± 0.05 (pseB), which albeit closer are still slightly below the cutoff of 0.25.

**Figure 7:**
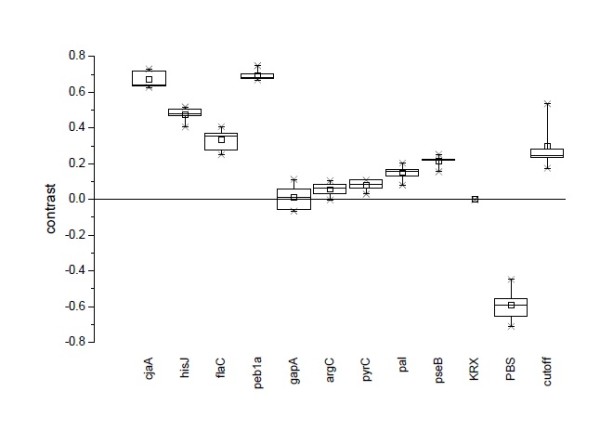
**Contrast values from all replicates**. The contrast values (n = 5) are presented as a box-whisker-plot where the square represents the arithmetic mean and the horizontal line the median. The whiskers enclose 90% of all values (95% - 5%) and 98% of the values fall between the crosses. CjaA, hisJ, flaC and peb1a show positive signals above the cutoff throughout the slides with only one exception for flaC due to an outlier in the cutoff. On the other hand, gapA, argC and pyrC show negative results, i.e. contrast values significantly below the respective cutoff values for all the slides. The remaining proteins pal and pseB are in the range of the cutoff values.

To validate the results from one slide technical replicates (n = 5) were analyzed. Summing up the results of all slides, a box-whisker-plot was composed, see Figure 7. The replicates emphasized the results from the one slide above. CjaA, hisJ, flaC and peb1a were clearly above the respective cutoffs and led to positive signals in all the slides. On the contrary, gapA, argC and pyrC were significantly below the cutoff. The tendency of pal and pseB to fall within close proximity of the cutoff is observed throughout the replicates.

**Figure 8:**
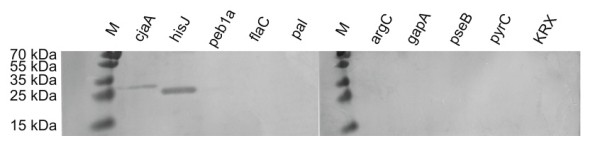
**Western Blot of purified protein**. CjaA and hisJ show strong bands of the corresponding sizes (31 kDa and 28 kDa) after removal of the HaloTag^®^. The remaining proteins cannot be detected in western blot by visual means.

**Figure 9:**
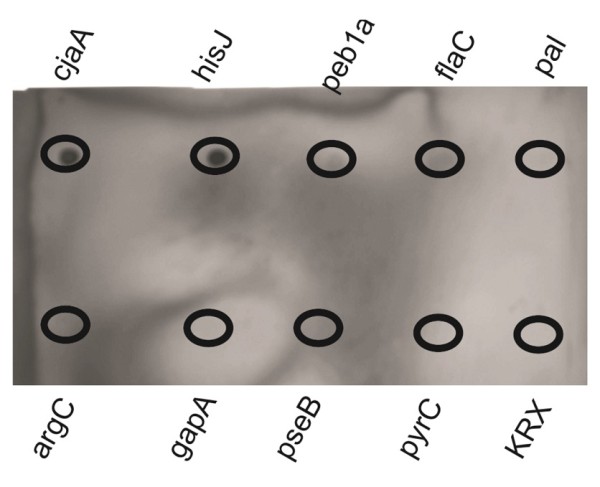
**Dot Blot of purified protein**. CjaA, hisJ and peb1a show positive signals, flaC and pal show extremely weak signals at best but are hardly distinguishable from the background. The rest is clearly negative showing no signals at all.

Further verification of the results was performed by using a standard western blot experiment to test for immunogenicity. Figure 8 shows the results of the investigated proteins after purification with HaloLink™ magnetic beads was performed prior to SDS-PAGE and blotting. Only two of the investigated immunogenic proteins (cjaA, and hisJ) show strong visible bands in western blot matching the expected sizes. The three remaining immunogenic proteins, peb1a, flaC and pal, cannot be distinguished in western blot analysis as well as all the other proteins. Additionally, a Dotblot was performed (Figure 9) with the purified proteins, which showed clear positive signals for cjaA and hisJ, a rather weak signal for peb1a and extremely low signals for flaC and pal.

**Figure 10:**
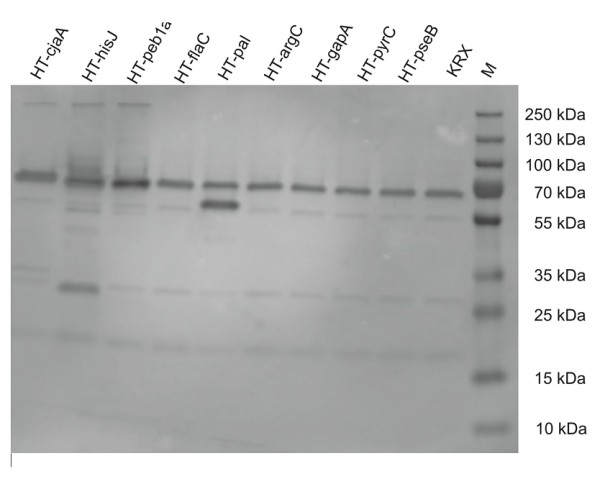
**Western Blot of whole cell lysates**. Four bands are visible in the KRX whole cell lysate with a more prominent band at 70 kDa and three weaker bands at 55 kDa, 28 kDa and roughly 18 kDa. These bands are most likely due to cross-reactive proteins within the *E.coli *lysates. All four proteins previously described as non-immunogenic (argC, gapA, pseB and pyrC) show band patterns highly similar to KRX. This underlines their lack of immunogenic potential. The five proteins previously described as immunogenic elsewhere show stronger bands at roughly 70 kDa for cjaA, hisJ, peb1a, flaC and 55 kDa for pal respectively. These sizes correspond closely to the expected sizes of the HaloTag^® ^fusion proteins. However, the interference of the background bands from KRX - being in the same range as the target proteins under investigations - prevents a secure identification of immunogenic proteins.

In contrast, Figure 10 shows a western blot performed directly with whole lysates after recombinant expression without further purification. At least four bands are visible in all the samples with the most prominent band at 70 kDa. Bands of lower intensity appear at approximately 55 kDa, 28 kDa and 18 kDa. The first five lanes, corresponding to the known immunogenic proteins, cjaA, hisJ, peb1a, flaC and pal, show bands of higher intensity than the remaining five lanes. However, as the investigated fusion proteins fall either into the 70 kDa or in case of HT-pal into the 55 kDa range, a clear differentiation between positive bands and background caused by KRX cross-reactive proteins is hardly possible.

## Discussion

Using our new method, we were able to express, immobilize and screen all of nine different proteins from *Campylobacter jejuni *using HaloTag^® ^and KRX cells. CjaA, hisJ, flaC, peb1A and pal act immunogenic as described elsewhere [18-23]. GapA, pyrC, argC and pseB have not been reported as immunogenic before. Immunoscreening on microarrays showed that gapA, pyrC and argC were significantly below the cutoff and can be considered negative.

Further, different codon usage between these two organisms (*Campylobacter jejuni *and *Escherichia coli*) might be the cause for low expression (see Additional file 1: Codonusage 1). This could have been a problem in the expression of pal and possibly to an extent for flaC explaining the low signals in microarray and more prominently in western blot analysis and dotblot. Nevertheless, four proteins with known immunogenic ability have been successfully detected with strong signals clearly above the cutoff.

So far, pseB has not been described as immunogenic, although it is involved in the glycosylation of the flagellar apparatus of *Campylobacter jejuni *[24,25]. Although the signals are far weaker than for known antigens such as cjaA, hisJ or peb1a, the results suggested immunogenic potential of pseB to be in the realm of possibility. The close proximity and involvement in the modification of flagellin, which is known to be immunogenic as well as highly exposed, suggests immunogenic behavior of pseB to be plausible. However, western blot and dot blot analyses have revealed no band or signal for pseB, which is in accordance to the microarray data. The contrast values were below the cutoff and no band was visible in western blot experiments.

Directly analyzing the whole lysates by western blot failed to discriminate between positive bands and background as the whole lysates of KRX cells clearly show cross-reactive signals with the used polyclonal antibody. However, as these bands bear similar sizes as the investigated fusion proteins, a secure identification of true positives is difficult at best using standard western blot without previous purification methods. Even then identification of immunogenicity is sometimes difficult at best as sensitivity might be low due to differences in expression rate and losses of protein content during purification. Still, the signal intensities of the five immunogenic proteins were well represented by all three methods - microarray, western blot and dot blot, as those with highest contrast values, namely cjaA, hisJ and peb1a, have also shown bands in western blot and signals in dot blot albeit lower for peb1a. In contrast, pal failed to generate a positive signal during microarray experiments and this result could be confirmed by western blot and dot blot. For flaC no band is visible in western blot and the signal in dot blot is extremely weak, yet our microarray method has identified the protein as being immunogenic, which has been reported elsewhere. A possible explanation for this is the high sensitivity of microarrays. This is well in accordance to Ekins "ambient analyte theory" [26] which has been the basic concept of microarrays for several decades. Thus, the microarray method might well be superior for immunoassays than common blotting methods.

## Conclusions

We were able to clone several genes from *Campylobacter jejuni *into KRX cells, to express the respective proteins as fusion constructs with a HaloTag^® ^attached to their N-Terminus and to immobilize these proteins on microarray surfaces. Subsequently, we have succeeded in screening the proteins using polyclonal antibodies to detect their immunogenic abilities. The usage of pFN18A Flexi vector in combination with KRX cells offers various advantages. First, Flexi vectors inhabit several intriguing features. They encode a bacterial RNase - Barnase - which is only removed during successful cloning. If cloning fails, the Barnase remains intact and leads to cell death and no visible colonies on the plates. The HaloTag^®^- a derivative of a Dehalogenase - is fused during expression to the N-terminus of our protein of interest. The expression of the fusion proteins is under control of T7 RNA Polymerase. Additionally, an ampicillin cassette is present to allow for antibiotic selection.

Second, with commonly used BL21(DE3) expression cells, induction is realized by isopropyl β-D-1-thiogalactopyranoside (IPTG). However, the IPTG-induced expression of T7 RNA Polymerase in BL21(DE3) is not tightly regulated, i.e. the promoter is leaky, causing a basal expression even if cells are not induced. This is a major problem especially if toxic substances are to be expressed [27]. In our method, we used KRX cells to counter this problem. These cells are under regulation of L-Rhamnose and induction can completely be turned off by addition of glucose to the medium during growth. In fact, for the proteins investigated, expression has failed in BL21(De3) for all but two proteins (data not shown), whereas KRX cells were able to express all the proteins with satisfying yields as we showed by SDS-PAGE.

Third, the HaloTag^® ^offers covalent binding to its specific ligand, therefore leading to a strong attachment of the proteins to the microarray surface in comparison to other tags such as GST or 6 × His. Expression, immobilization to modified surfaces and detection of antigens was difficult at best using 6 × His and GST (data not shown).

Combining these features with microarrays, we have been able to employ a method to rapidly screen for immunogenic proteins without the need for an additional purification step. Instead whole lysates can directly be spotted to the microarray surface. Still, cross-reactivity is prevented by the special use of the Tag and its specific ligand on the microarray slide. Further, the use of microarrays as a screening platform offers several advantages in itself. High throughput, statistical testing of the data and numerous replicates are easily achieved compared to the more traditional screening on nitrocellulose membranes.

In fact, numerous replications with nitrocellulose membranes are difficult, the selection of positive clones error-prone as replicate and master filters have to be compared visually and selection occurs manually. Moreover, nitrocellulose membranes bind entire protein lysates unable to discriminate between proteins of interest and proteins from the expression host. This high background content leads to numerous false positive signals when using polyclonal sera raised against whole cells to search for new antigens. Although, these effects might differ for different bacteria, in case of *Campylobacter jejuni *and *Escherichia coli *this has been observed previously as their outer membrane proteins (OMP) show high similarities [28]. On top of that, identification of positive clones is merely done visually, usually by chemiluminescence.

With our method, these problems are minimized. It allows screening of numerous clones on microarrays with sufficient replicates to gain statistically significant data. It reduces the occurrence of false positive signals to a minimum, thus focusing on the real positive proteins and leading to a faster detection. It allows for statistical and mathematical analysis of the data compared to nitrocellulose membranes illuminating proteins with different strength in their immunogenic ability. Further, the choice of our expression host - KRX - and the HaloTag^® ^have brought other benefits, as well. The expression is tightly regulated, offers high yields and the covalent attachment of the proteins to the surface makes separate purification obsolete as it combines spotting and purification directly. The overall time frame from cultivation to screening is roughly 30 hours, whereas immunoscreening on nitrocellulose membranes [8] takes at least 3 days. The benefit of low turn-around time increases, when taking the cloning into consideration. Normally, cloning to recombinant expressed proteins is first done in propagation cells such as JM109 or DH5α and afterwards a second cloning procedure to expression cells as BL21(DE3) is performed. With KRX single-step cells both propagation and expression can be performed sequentially avoiding time-consuming plasmid preparations and additional cloning steps.

Our results proved to be reproducible, which we showed using several technical replicates.

The HaloTag^® ^has previously been used for an increasing spectrum of applications, such as the monitoring of single molecules during trans-translation processes [29], reporter protein assays for magnetic resonance imaging (MRI) analysis [30] and flow cytometry [31]. With our method, we have added another intriguing application to the mix. This method ought to become an attractive alternative to traditional methods for immunoscreening as it features several unique advantages. It provides a low turn-around time, significantly reduces background and false positive signals and enables high-throughput screenings of expression libraries or other sources of hundreds and thousands of different clones. With our results we were able to show the great potential of this method in future full proteome screenings as an attractive alternative to other broad screenings. Its major advantages being the complete deletion of cross-reactivity to proteins of the expression host as these are simply washed off after spotting. Thus an additional purification step, which is usually performed in microarray-based screening approaches of expression libraries or proteome analysis before immobilization to Nitrocellulose slides [32], becomes obsolete. Therefore, this method might decrease time and material costs associated with extensive screenings of libraries. These advantages increase significantly with the number of different samples processed, making library screening a preferred application.

For screening of whole expression libraries and the detection of novel antigens the use of different polyclonal sera and antibodies is necessary as these might detect different antigens. Only if a protein leads to positive signals in all screenings it can be considered immunogenic. Additionally, controls using established methods such as western blot, immunoprecipitation and ELISA ought to be considered to validate the potential of a novel antigen.

Consequently, the method presented herein may be useful for broad screening of bacterial expression libraries to identify and detect novel immunogenic proteins. Our approach gives the possibility to discover new proteins that could be used as biomarkers in diagnostic assays or for the production of vaccines. Additionally, further knowledge about potentially new virulence-associated factors and antigens will grant deeper insight and understanding of virulence and pathogenicity of many enigmatic bacteria. Therefore, we strongly believe this to emerge as an important tool in the search for antigens, virulence factors, biomarkers and vaccine candidates.

## Methods

### Bacterial cultivation

*Campylobacter jejuni *NCTC 11168 was used for isolation of genomic DNA and amplification of genes used in this study. *Campylobacter jejuni *NCTC 11168 was streaked on Karmali Agar (Oxoid) and incubated under microaerophilic conditions at 42°C for at least 2 days. For genomic DNA isolation a flask of 100 ml brain-heart-infusion (BHI) broth was inoculated with a loop of bacterial culture and cultivated in an Innova 40R (New Brunswick) incubation shaker for 48 h at 42°C in an anaerobic jar.

### Genomic DNA isolation

Genomic DNA of *Campylobacter jejuni *NCTC 11168 was isolated using a phenol-chloroform protocol. Briefly, cells were harvested by centrifugation (1000 g, 10 minutes) in a benchtop centrifuge 5415 R (Eppendorf) and the pellet resuspended in 1 ml of 1 × PBS (1.9 mM NaH_2_PO_4_, 8.1 mM Na_2_HPO_4_, 150 mM NaCl). After a second centrifugation the pellet was resuspended in 500 μl of lysis buffer (10 mM Tris, pH 7.5, 25 mM EDTA, 75 mM NaCl, 0.1% SDS and 0.5 mg/ml Proteinase K) and incubated for 1 h at 50°C. Afterwards one volume of phenol-chloroform-isoamyl alcohol (25:24:1) was added and after centrifugation (> 13.000 g, 1 min) the DNA-containing top phase was carefully removed and transferred to a clean tube. This step was repeated three times and the combined top phases washed twice with chloroform-isoamyl alcohol (24:1). After addition of 1/10 volume of 3 M sodium acetate and one volume of 100% isopropanol the solution was centrifuged for 30 min at 13.000 g. The supernatant was discarded, the pellet air-dried and resuspended in 100 μl Tris-Cl (pH 8.5). The concentration and purity of the isolated DNA was measured using the Nanodrop ND-1000 device (Peqlab).

### Amplification of genes of interest

PCR was carried out to selectively amplify several genes of interest from the genomic DNA. The primers were designed using Flexi Vector Primer Design Tool (Promega) and possessed overhangs for subsequent cloning into Flexi Vectors (Promega). The melting temperature of each primer was determined using a T_m _calculator (Finnzymes). Table 2 summarizes primer sequences and melting points.

**Table 2:** Overview of genes investigated.

Gene	Primer	Sequence (5'-3')	T_m _[°C]	Length [bp]	immunogenic
*cjaA *[EMBL: CAL35100]	cjaA for	TAAAGCGATCGCCATGAAAAAAATACTTCTAAGTGT	53	840	+
			
	cjaA rev	ATTAGTTTAAACAATTTTTCCACCTTCAATCA	59		

*hisJ *[EMBL: CAL34871]	hisJ for	GGCTGCGATCGCCATGAAAAAAATATTAAGCATTGCTC	61	756	+
			
	hisJ rev	CAAAGTTTAAACTTCTAATTCATATTTTTTAATTAAAGTA	55		

*pal *[EMBL: CAL34284]	pal for	ATATGCGATCGCCATGAAAAAAATTCTTTTTACTTC	55	498	+
			
	pal rev	GCACGTTTAAACTCTTGATAATTTAAATTCAGC	54		

*cfrA *[EMBL: CAL34884]	cfrA for	TCAGGCGATCGCCATGAAAAAAATATGTCTATCAG	52	2091	+
			
	cfrA rev	GATGGTTTAAACAAAGTTACCATTGATAGAAATA	52		

*flaC *[EMBL: CAL34857]	flaC for	TGTCGCGATCGCCATGATGATCTCTGATGCAACT	59	750	+
			
	flaC rev	GGGGGTTTAAACTTGTAATAAATTAGCAATTTTGCTT	59		

*flaA *[EMBL: CAL35451]	flaA for	AACTGCGATCGCCATGGGATTTCGTATTAACACC	60	1719	+
			
	flaA rev	CCGTGTTTAAACCTGTAGTAATCTTAAAACATTTTG	54		

*peb1a *[EMBL: CAL35041]	peb1 for	TAAAGCGATCGCCATGGTTTTTAGAAAATCTTTGT	56	780	+
			
	peb1 rev	AGGGGTTTAAACTAAACCCCATTTTTTCGCTA	61		

*cadF *[EMBL: CAL35585]	cadF for	TAAAGCGATCGCCATGAAAAAAATATTCTTATGT	50	960	+
			
	cadF rev	TGTCGTTTAAACTCTTAAAATAAATTTAGCAT	49		

*pyrC *[EMBL: CAL34413]	pyrC for	GCAGGCGATCGCCATGAAACTTAAAAATCCTTTAG	53	1008	-
			
	pyrC rev	ACGTGTTTAAACATGTTTTAATTGAAATTTCAAA	55		

*pseB *[EMBL: CAL35407]	pseB for	GACCGCGATCGCCATGTTTAACAAAAAAAATATCT	52	1005	-
			
	pseB rev	TTCTGTTTAAACAAAACCTTCAGTATGATTGAT	55		

*gapA *[EMBL: CAL35512]	gapA for	AACAGCGATCGCCATGGCTGTAAAAGTTGCTATAAATGGT	65	999	-
			
	gapA rev	CACAGTTTAAACAGCCTTATTTGCAATATATACTGCCA	65		

*argC *[EMBL: CAL34379]	argC for	GTTCGCGATCGCCATGAAAATAAAAGTTGGGATTTTAG	60	1028	-
			
	argC rev	CTTGGTTTAAACCAAATTTGCAAAGAATTTAAGTC	59		

The gene name, accession from EMBL, primer names and sequences, their melting points, length of gene and immunogenic characteristics of the encoded protein are given

The amplification was performed in separate tubes containing a reaction mixture comprised of 2 μl DNA (200 ng), 10 μl 5× Phusion HF buffer, 1 μl dNTPs (10 mM each), 0.5 μl 50 mM MgCl_2 _(final concentration 2 mM) 5 μl (10 μM) of each primer, 0.5 μl Phusion High Fidelity Polymerase (2 U/μl, Finnzymes) and PCR-grade sterile water to a final volume of 50 μl. Amplification of the targets was performed in a gradient Thermocycler (Biorad): initial denaturation 98°C for 3 min, 30 cycles of 98°C for 10 s, annealing temperature (T_m _+ 3°C of the lower primer) for 30 s and 72°C for 30 s and a final extension of 72°C for 3 min. The PCR products were purified using QIAquick PCR Purification Kit (Qiagen) according to the manufacturer's instructions. The concentration and purity of DNA were measured with the Nanodrop device and the size of the amplicon determined by using an agarose gel electrophoresis.

Agarose gel electrophoresis was carried out in a PerfectBlue Gelsystem Mini (Peqlab) for 90 min at 80 V. The DNA was visualized by ethidium bromide staining and detected on a transilluminator. Hyper Ladder I (Bioline) served as a size reference.

### Ligation and cloning

After the correct size was determined the purified PCR product was used in restriction digest according to the Flexi Vector System (Promega). Briefly, the DNA and the acceptor vector, Flexi pFN18A (Promega) were cut by *Sgf*I and *Pme*I. For the reactions 500 ng of DNA were mixed with 4 μl 5× Flexi Digest buffer, 4 μl of Flexi Enzyme Blend (*Sgf*I and *Pme*I) and the total volume adjusted to 20 μl using nuclease-free water. Respectively, 200 ng of acceptor vector were cut after combining it with 4 μl of 5× Flexi Digest buffer, 2 μl of Flexi Enzyme Blend (*Sgf*I and *Pme*I) and 12 μl of nuclease-free water. The reactions were incubated for 30 min at 37°C. Afterwards, the reactions containing the different amplified genes were purified by QIAquick PCR Purification Kit (Qiagen), whereas the digest of vector was heated for 20 min to 65°C to inactivate both enzymes. Directly after purification and inactivation 4 μl of restricted DNA was added to 10 μl 2× ligase buffer, 5 μl of digested acceptor vector and 1 μl T4 DNA Ligase (HC, 20 U/μl). The ligation reaction proceeded for one hour at room temperature.

The ligated constructs of vector and gene of interest were cloned into KRX Single-Step Competent Cells (Promega) by following the manufacturer's instructions. 200 μl of each transformation reaction were spread on LB Agar plates containing ampicillin (100 μg/ml). The plates were incubated overnight at 37°C in an incubator.

### Selection of positive clones

From each plate seven clones were selected and used as templates in colony PCR. The reaction mixture contained 11.2 μl nuclease-free water, 4 μl 5× Phusion HF buffer, 0.4 μl dNTPs (10 mM each), 0.2 μl 50 mM MgCl_2 _(final concentration 2 mM), 2 μl of each Primer (10 μM) and 0.2 μl of Phusion High Fidelity Polymerase. The colony PCR and gel electrophoresis conditions were identical to the ones described above.

Positive clones were used to inoculate 5 ml of lysogeny broth containing ampicillin (LB-amp) and cultivated under shaking conditions (270 rpm) at 37°C, until an optical density of OD_600 _= 0.6 was reached. The cell suspension was centrifuged (1000 g, 5 min). The supernatant was discarded and the pellet resuspended in 0.9 ml of fresh LB-amp. Subsequently 100 μl sterile DMSO (Roth) was added. Clones were stored in cryo tubes at -80°C.

### Protein expression, lysis and spotting of lysates to HaloLink™ Slides

For protein expression, clones from cryo stocks were used to inoculate 5 ml of LB-Amp and incubated for 8 h at 37°C in an incubation shaker. The temperature and shaking were decreased to 18°C respectively 180 rpm. Protein expression was induced by addition of 25 μl of 20% sterile-filtrated L-Rhamnose (Promega). The incubation continued overnight. Lysis of cells was achieved using EasyLyse Bacterial Protein Extraction Kit (Epicentre) according to manufacterer's instructions. Afterwards lysates were spotted to HaloLink™ Slides (Promega) using the QArray2 microarray spotter (Molecular Devices). Humidity was set to 70% during spotting and the slides were left in the humidity chamber for 1 h after spotting was completed.

### Immunoscreening on HaloLink™ Slides

The slides were washed after spotting with PBSI (1× PBS, 0.05% IGEPAL^® ^CA-630) and a 3 Well ProPlate™ module (Grace Biolabs) was attached to each slide. Each chamber was filled differently, as follows: Top chamber with rabbit-polyclonal antibody to *Campylobacter jejuni *(Acris, 5 μg/ml), middle chamber with rabbit-polyclonal to HaloTag^® ^(Promega, 5 μg/ml) and bottom chamber with PBS only. Incubation was performed for one hour at room temperature under gentle rocking. The slides were washed with PBSI. Secondary antibody (goat polyclonal to rabbit IgG conjugated with Chromeo™-547, Abcam, 10 μg/ml) was applied to each chamber. The slides were incubated under gentle rocking for one hour at room temperature in the dark. Finally, slides were washed with PBSI, the ProPlate™ module was removed and the slides were dried by a nitrogen stream. The scanning of each slide was performed on an Axon Genepix 4200A laser scanner (Molecular Devices) with the following settings: 532 nm laser, PMT 400, 40% power, lines to average 1, 10 μm resolution, standard green emission filter 575 nm.

### Microarray analysis

The raw data of the microarray experiment [GEO: GSE33295] was analyzed using OriginPro 8 G (OriginLab). The local background of each spot was subtracted from each spot's intensity to gain relative fluorescence intensities (RFI). The signals from chamber one were corrected through substraction of RFIs of chamber three to account for unspecific signals by secondary antibody immobilization alone. From the KRX extract samples without HaloTag^® ^protein the median and the standard deviation (SD) of the background are calculated. For each sample the contrast is computed by:

$$c=\frac{\left[RFI_{s}-median\left(b\right)\right]}{\left[RFI_{s}+median\left(b\right)\right]}$$

with RFI_S _the relative fluorescence intensity of each spot and the median of the background median(b). As samples were spotted in multiple replicates, mean contrast values and their respective standard deviations were calculated. The cutoff was calculated using the median and SD of the background:

$$cutoff=\frac{\left[\left(median\left(b\right)+5\times SD\left(b\right)-median\left(b\right)\right)\right]}{\left[\left(median\left(b\right)+5\times SD\left(b\right)+median\left(b\right)\right)\right]}$$

### Sequencing

Plasmids were isolated from 1 ml aliquots of uninduced cells by QIAprep Spin Miniprep Kit (Qiagen) according to the manufacturer's instructions. The purified plasmid DNA was eluted in 50 μl Tris-HCl (pH 8.5). Concentration and purity were determined by Nanodrop measurements. Each plasmid was sent to Sequence Laboratories Göttingen GmbH for sequencing using two different sequencing primers HT7 F (5' acatcggcccgggtctgaatc 3') and FLX R (5' cttcctttcgggctttgttag 3'). The generated sequences were aligned with the corresponding gene sequences by global alignment with free-end gaps (modified Needleman-Wunsch) using Geneious Pro™ 5.4.4 (Biomatters).

### SDS-PAGE

The expression of the desired HaloTag^® ^fusion proteins was checked by SDS-PAGE. After lysis of cells, 2 μl of each protein extract was mixed with 1 μl of 10 μM HaloTag^® ^Alexa 488 ligand. After addition of 7 μL 1× TBS (100 mM Tris, 150 mM NaCl, pH 7.6) the reaction was incubated at room temperature for 30 minutes. 2 μl of each reaction were removed, mixed with 8 μl of 5× loading buffer (Fermentas) and 1 μl DTT and heated for 5 min at 70°C. The separation was performed on a Mini-Protean TGX Gel (Biorad, any kD, 15 wells) in a Protean II xi Cell chamber (Biorad) for 30 min at 200 V. As a size reference PageRuler Plus prestained protein ladder (Fermentas) was used in combination with HaloTag^® ^standard protein (Promega). Fluorescence was measured in a FLA-5100 (Fujifilm) with excitation at 473 nm.

### Western Blot Analysis

For western blot analysis the samples were run on an SDS-PAGE, the Gel blotted to a PVDF membrane using the iBlot^® ^Western Blotting System (Life Technologies). The blotting time was set to 7 minutes. After blotting, membranes were rinsed with water and blocked for 1 h in 5% fat-free milk powder in PBS with gentle rocking. The membranes were then washed with PBS-Tween (0.05%) twice for five minutes each. The primary antibody (4 μg/ml) was added in 1% fat-free milk powder in PBS and incubation was performed for 1.5 h under gentle rocking. Afterwards, membranes were washed twice with PBS-Tween (0.05%). The AP-conjugated secondary antibodies (4 μg/ml) were added in 1% fat-free milk powder in PBS and incubation proceeded for 1 h under gentle rocking. Finally, membranes were washed twice with PBS-Tween and Western Blue^® ^stabilized substrate for Alkaline Phosphatase (Promega) was added. After 5 min the membrane was taken out, rinsed with water and dried. A picture of the membrane was taken using BioDocAnalyze digital and its software (Biometra).

### Dot Blot

For dot blot analysis, 2 μl of the purified protein solution were added as small droplets to a nitrocellulose membrane. After drying, the membrane was blocked using 5% fat-free milk powder in PBS for 1 h. The membrane was washed twice with PBS-Tween (0.05%) and primary antibody (4 μg/ml) was added in 1% fat-free milk powder in PBS. Incubation prolonged for 30 min with gentle rocking, afterwards the membrane was washed twice as above and AP-conjugated secondary antibody (4 μg/ml) was added and incubation proceeded for additional 30 min. After a final two washes, the Western Blue^® ^stabilized substrate for Alkaline Phosphatase (Promega) was added and after 5 min the membrane was rinsed with water. The visualization was performed with the BioDocAnalyze digital software.

### Protein purification

The recombinantly expressed target-proteins were purified from whole lysates by using HaloLink™ magnetic beads (Promega) according to the manufacturer's instructions. Subsequently, target protein was cleaved off using ProTEV Plus Protease and the supernatant used for SDS-PAGE.

## Competing interests

The authors declare that they have no competing interests.

## Authors' contributions

SH carried out the design of the method, the testing of the method including all cloning steps, interpretation of sequence data, data analysis and drafted the manuscript. MvNR conceived and coordinated the study, participated in the analysis of the data and helped draft the manuscript. FFB participated in the critical review and drafting of the manuscript. All authors read and approved the final manuscript.

## Supplementary Material

Additional file 1 **Excel sheet comparing the codon usage of *Campylobacter jejuni *NCTC 11168 and *Escherichia coli *strain K12**. Click here for file
